# Carnosine Supplementation Improves Serum Resistin Concentrations in Overweight or Obese Otherwise Healthy Adults: A Pilot Randomized Trial

**DOI:** 10.3390/nu10091258

**Published:** 2018-09-07

**Authors:** Estifanos Baye, Jozef Ukropec, Maximilian P. J. de Courten, Aya Mousa, Timea Kurdiova, Josphin Johnson, Kirsty Wilson, Magdalena Plebanski, Giancarlo Aldini, Barbara Ukropcova, Barbora de Courten

**Affiliations:** 1 Monash Centre for Health Research and Implementation, School of Public Health and Preventive Medicine, Monash University, Melbourne, Victoria 3168, Australia; estifanos.baye@monash.edu (E.B.); aya.mousa@monash.edu (A.M.); josphin.johnson@monash.edu (J.J.); 2 Institute of Experimental Endocrinology, Biomedical Research Centre, Slovak Academy of Sciences, 84236 Bratislava, Slovakia; jozef.ukropec@gmail.com (J.U.); timea.kurdiova@savba.sk (T.K.); barbara.ukropcova@gmail.com (B.U.); 3 Centre for Chronic Disease, College of Health and Biomedicine, Victoria University, Melbourne, Victoria 3800, Australia; Maximilian.deCourten@vu.edu.au; 4 Department of Immunology and Pathology, Monash University, Melbourne, Victoria 3168, Australia; kirsty.wilson@monash.edu (K.W.); magdalena.plebanski@monash.edu (M.P.); 5 School of Health and Biomedical Sciences, RMIT University, Melbourne, Victoria 3001, Australia; 6 Department of Pharmaceutical Sciences, Università degli Studi di Milano, 20133 Milan, Italy; giancarlo.aldini@unimi.it; 7 Institute of Pathological Physiology, Faculty of Medicine, Comenius University, 84215 Bratislava, Slovakia

**Keywords:** carnosine, adipokines, obesity, type 2 diabetes, cardiovascular disease

## Abstract

Adipokines play an important role in the regulation of glucose metabolism. We have previously shown that carnosine supplementation in overweight or obese non-diabetic individuals improves glucose metabolism but does not change adiponectin concentrations. However, its effect on other adipokines has not been investigated. Herein we further determined the effect of carnosine supplementation on serum adipsin, resistin and leptin. Twenty-two overweight or obese otherwise healthy adults were randomly assigned to receive either 2 g of carnosine (*n* = 13) or identically looking placebo (*n* = 9) for 12 weeks. Serum adipsin, leptin and resistin were analyzed using a bead-based multiplex assay. Carnosine supplementation decreased serum resistin concentrations compared to placebo (mean change from baseline: −35 ± 83 carnosine vs. 35 ± 55 ng/mL placebo, *p* = 0.04). There was a trend for a reduction in serum leptin concentrations after carnosine supplementation (−76 ± 165 ng/mL carnosine vs. 20 ± 28 ng/mL placebo, *p* = 0.06). The changes in leptin and resistin concentrations were inversely related to the change in concentration for urinary carnosine (*r* = −0.72, *p* = 0.0002; *r* = −0.67, *p* = 0.0009, respectively), carnosine-propanal (*r* = −0.56, *p* = 0.005; *r* = −0.63, *p* = 0.001, respectively) and carnosine-propanol (*r* = −0.61, *p* = 0.002; *r* = −0.60, *p* = 0.002, respectively). There were no differences between groups in change in adipsin concentrations. Our findings show carnosine supplementation may normalize some, but not all, of the serum adipokine concentrations involved in glucose metabolism, in overweight and obese individuals. Further clinical trials with larger samples are needed to confirm these results.

## 1. Introduction

Adipokines are bioactive peptides that are secreted by adipose tissue [1]. Disruption of normal adipose tissue function due to excess abdominal fat accumulation, as observed in obesity, leads to dysregulated production of adipokines [1,2]. Consequently, concentrations of adipokines in the bloodstream are altered. Although the exact mechanisms remain unclear, altered adipokines concentrations have been shown to induce inflammation and impair glucose and lipid metabolism [3,4], thereby contributing to the development of obesity-related chronic diseases including cardiometabolic [5,6] and neurodegenerative diseases [7]. Adipokines have been shown to have systemic effects on target organs important for glucose metabolism and heart disease [8], including the brain, heart, muscle, liver, and pancreas [6], and also have a role in the regulation of appetite, satiety, and energy expenditure [4]. Adipokines have been proposed not only as novel biomarkers but also as regulators or potential targets of cardiometabolic and other chronic diseases [4,9]. Therefore, interventions which target adipokines may be beneficial in the treatment of obesity-related chronic diseases [4].

Carnosine, an over-the-counter food supplement composed of β-alanine and L-histidine molecules, has been shown to ameliorate cardiometabolic risk factors and diseases by reducing inflammation, oxidative stress, and advanced glycation, as demonstrated by several animal studies [10,11,12,13,14]. Supplementation with carnosine has therefore been suggested as a potential strategy for prevention and treatment of cardiometabolic disease [15,16,17]. However, there is little evidence from human studies examining the efficacy of carnosine supplementation in reducing cardiometabolic risk factors [16,17,18,19]. We have previously shown that carnosine supplementation hampered an increase in insulin resistance and improved glucose tolerance, but had no effect on circulating adiponectin concentrations [16]. To our knowledge, the effects of carnosine on other adipokines have not previously been investigated. Therefore, we aimed to address this knowledge gap by examining whether, in a high-risk group of overweight or obese sedentary individuals, supplementation with carnosine would reduce serum resistin, leptin, and adipsin concentrations, all of which have been associated with chronic low-grade inflammation and cardiometabolic diseases [9].

## 2. Materials and Methods

### 2.1. Study Design and Participants

This study is a secondary analysis of data from a pilot randomized, double-blind, placebo-controlled trial which was performed in 30 overweight and obese sedentary individuals. The trial was conducted at the Institute of Experimental Endocrinology, Slovak Academy of Sciences, Slovakia, and detailed study methods as well as the primary outcomes have been published [16]. Briefly, study participants were recruited from the community in Bratislava, Slovakia from September to October 2013 via newspaper advertisements. Participants were enrolled in the study if they were non-smokers and did not use illicit drugs or take any medications or food supplements, and were non-diabetic based on a 75 g oral glucose tolerance test (OGTT). All participants were overweight or obese (body mass index (BMI) ≥ 25 kg/m^2^) and did not participate in any regular structured physical activity, but otherwise healthy based on physical examination and routine blood analyses. All participants were advised to maintain their usual diet and exercise habits and those who had a weight change ≥5 kg over the 12 week study period were excluded. Participants with clinical or laboratory signs of infection or acute inflammation were excluded for the purpose of this analysis.

The study protocol conforms to principles of the Declaration of Helsinki and was approved by the Ethics Committee of the University Hospital Bratislava, Comenius University, Bratislava, Slovakia. All participants provided written informed consent prior to participation.

### 2.2. Intervention and Outcome Measures

Participants were randomly allocated to receive either 1 g carnosine twice daily (Flamma S.p.A, Bergamo, Italy) or matching placebo (1 g sucrose) for 12 weeks. Participants, investigators, and outcome assessors were blinded to group assignment until after primary data analysis was complete. Eligible participants underwent a study protocol which has been described in detail [16]. In brief, the protocol included anthropometric assessments, blood pressure and lipid profile measurements, OGTT, and assessment of biochemical parameters including urinary carnosine, carnosinase-1 content and activity, high-sensitivity C-reactive protein (hsCRP) and circulating serum adipokine concentrations including leptin, resistin, and adipsin. Prior to metabolic testing, participants were asked to refrain from strenuous exercise and caffeine for 3 days. All metabolic testing and blood and urine collections were performed under blinded conditions after a 12 h overnight fast (with no carnosine ingestion for 12 h).

### 2.3. Anthropometric Measurements

Body weight and height were measured and used to calculate BMI (weight (kg)/square of height (m)). Waist circumference was measured at the midpoint between the lower border of the rib cage and the iliac crest in the horizontal plane. While the subjects were standing, hip circumference was measured at the point yielding the maximum circumference over the buttocks. These measurements were used to calculate waist-to-hip ratio as an additional index for body fat distribution (waist (cm)/hip (cm) = waist-to-hip ratio).

### 2.4. Measurement of Adipokines and hsCRP

Serum adipokines including adipsin, leptin, and resistin were quantified simultaneously by a bead-based multiplex assay (LEGENDplex™ Human Metabolic Panel, Cat. No. 740212, BioLegend, San Diego, CA, USA). This assay uses distinguishing bead populations which bind to the specified analyte, and are differentiated by a LSRII Fortessa flow cytometer (Becton Dickinson (BD), San Diego, CA, USA). Serum samples were diluted 200-fold as per manufacturer’s instructions and based on preliminary testing. Standards and serum samples were mixed with sonicated pre-mixed beads and detection antibodies in a 96-well v-bottom plate and incubated in the dark on a plate shaker (600 rpm, 2 h, room temperature (RT)). Streptavidin–phycoerythrin (SA-PE) conjugate was added to each well, and the plate was further incubated in the dark on a plate shaker (600 rpm, 30 min, RT). The beads were centrifuged and pelleted, and further re-suspended in wash buffer. The samples were transferred to micro FACS tubes and read on a LSRII flow cytometer (BD, San Diego, CA, USA) and analyzed using FACS DIVA software (BD, San Diego, CA ,USA). Data were analyzed with the provided LegendPlex™ Data Analysis Software (BioLegend, San Diego, CA, USA) with standard curves generated from 0 to 200,000 pg/mL and samples adjusted for dilution factors. Intra- and inter-assay coefficients of variation (CVs) for the analytes were <7%. Serum hsCRP was measured by an immunoturbidimetric assay (Randox, UK). Intra- and inter-assay CVs for hsCRP were <10%.

### 2.5. Measurement of Urinary Carnosine and Carnosine Adducts, and Serum Carnosinase Content and Activity

Carnosine and carnosine adducts were measured in urine using an internal standard and a triple quadrupole mass spectrometer (TSQ Quantum Ultra, Thermo Scientific, Rodano, Italy) [20]. Carnosinase activity in serum was quantified by fluorometric determination of liberated histidine after carnosine addition), as previously reported [16]. Serum carnosinase content was measured using a sandwich enzyme-linked immunosorbent assay (ELISA) developed by Adelmann et al. [21], as per instructions.

### 2.6. Statistical Analysis

The sample size calculation has been reported elsewhere [16], and was based on the primary outcome of insulin sensitivity. Results are reported as means and standard deviations unless otherwise specified. Independent Student’s *t*-tests or Mann–Whitney tests were used to compare participants’ baseline characteristics between groups for normally and non-normally distributed variables, respectively. Due to the skewed distributions of the adipokines, all results for the adipokines analyses were assessed using non-parametric tests. Spearman correlations were used to examine relationships between change in adipokines and change in carnosine concentrations in the entire sample. Quantile regression analyses were performed to determine the associations between adipokines and urinary carnosine measurements after adjusting for predetermined clinically relevant factors including age, sex, change in BMI, and intervention group. Change in outcomes within each group (within-group difference from baseline to follow up) was assessed using Wilcoxon signed rank tests. Differences in change values between groups were determined using Mann–Whitney tests. Data analyses were performed using Stata V.14 (StatCorp, LP, College Station, TX, USA) and a two-sided *p* < 0.05 was considered statistically significant.

## 3. Results

Of the thirty participants included in the study, three (1 carnosine, 2 placebo) were excluded due to non-compliance with the protocol [16]. An additional five participants (2 carnosine, 3 placebo), had unusually high concentrations of plasma inflammatory markers, as measured by hsCRP, and hence were also excluded. The remaining twenty-two participants were included in the analysis, with 13 (10 M/3 F) in the carnosine group and 9 (8 M/1 F) in placebo. The mean age ± standard deviation of participants was 43.4 ± 8.1 years with no significant difference between groups (*p* = 0.7). There were no differences between groups in baseline anthropometric measures including BMI (31.03 ± 4.21 kg/m^2^ carnosine vs. 31.45 ± 3.62 kg/m^2^ placebo, *p* = 0.8) and waist-to-hip ratio (0.89 ± 0.06 carnosine vs. 0.89 ± 0.07 placebo, *p* = 0.8). No differences between carnosine and placebo groups were observed for baseline cardiometabolic and carnosine measures (all *p* > 0.06) or for serum adipsin, leptin, and resistin concentrations (Table 1).

### 3.1. Effect of Carnosine Supplementation on Serum Adipokines

Change in serum resistin concentrations were significantly different between the carnosine and placebo groups (mean change difference: −56.15 ± 29.08 ng/mL, *p* = 0.04), indicating a reduction of resistin in the carnosine-treated individuals. A trend for reduced leptin concentrations was observed after carnosine supplementation compared to placebo (mean change difference: −111.74 ± 57.66 ng/mL, *p* = 0.06). There were no differences in change in adipsin concentrations between the groups receiving carnosine and those receiving placebo (Table 1).

### 3.2. Associations between Carnosine Variables and Serum Adipokine Concentrations

Changes in both leptin and resistin concentrations were inversely correlated with a change in urinary carnosine (*r* = −0.75, *p* = 0.001; *r* = −0.71, *p* = 0.0001, respectively), carnosine-propanal (*r* = −0.56, *p* = 0.005; *r* = −0.63, *p* = 0.002, respectively) and carnosine-propanol concentrations (*r* = −0.61, *p* = 0.002; *r* = −0.60, *p* = 0.002, respectively). Change in carnosinase-1 content or activity were not correlated with either change in leptin or resistin concentrations (both *p* > 0.1).

Results of the median regression analyses are presented in Table 2. After adjustment for age, sex, change in BMI, and intervention group, the observed associations for change in urinary carnosine and carnosine-propanol with change in leptin and resistin remained significant (all *p* < 0.01; Table 2). Similarly, carnosine-propanal remained associated with leptin concentrations (*p* = 0.01; Table 2); however, the association with resistin was not longer significant (*p* = 0.1; Table 2).

## 4. Discussion

To the best of our knowledge, this is the first study examining the effects of carnosine supplementation on serum resistin, leptin, and adipsin concentrations in healthy humans. We found that carnosine supplementation reduced serum resistin and leptin concentrations in overweight or obese non-diabetic adults, and changes in serum resistin and leptin concentrations were inversely associated with changes in urinary carnosine and carnosine adducts.

We report that carnosine supplementation reduced serum resistin and leptin concentrations compared to placebo, and changes in urinary carnosine and carnosine adducts were inversely associated with changes in leptin and resistin concentrations. To our knowledge, no previous animal or human studies have examined relationships between carnosine and resistin or adipsin, nor the effects of carnosine supplementation on these adipokines. Both resistin and adipsin have been implicated in chronic low-grade inflammation and in the development of type 2 diabetes and cardiovascular disease [22,23,24], hence further studies investigating the effects of carnosine on these adipokines are warranted. Similarly, only one study examined the effects of carnosine on leptin [17], and did not show any change in leptin concentrations. However, this study used a supplement which was a combination of carnosine with cinnamon and chromium—some of which may have been biologically active [17]. In addition, the previous study [17] involved prediabetic, overweight and obese individuals who were less obese than our cohort (average weight difference of more than 10 kg). Consistent with this, baseline leptin concentrations in the previous study were lower (Mean= 31.0 ± 21.6 ng/mL in the intervention group) compared to a baseline leptin concentration of 115 ± 146.5 ng/mL in the carnosine group in our study, indicating a potentially higher degree of leptin resistance in our study participants. It is therefore possible that carnosine supplementation may be more effective in reducing leptin concentrations in the presence of greater obesity and higher leptin concentrations; however, further studies are needed to confirm this.

There are several mechanisms by which carnosine supplementation may affect concentrations of adipokines such as leptin and resistin, as was observed in our study. One potential mechanism occurs via the effects of carnosine on oxidative stress. Carnosine has been shown to reduce systemic and tissue oxidative stress by reducing carbonylated proteins and advanced lipoxidation end products (ALEs), decreasing the activity of superoxide dismutase and increasing the activity of liver enzymes [25,26,27]. These oxidative stress pathways have been shown to affect the synthesis and secretion of adipokines. For instance, positive associations between leptin and markers of oxidative stress have been reported in healthy middle-aged women and in non-diabetic hypercholesteremic patients [28,29]. Similarly, in individuals with both obesity and type 2 diabetes, leptin was positively correlated with lipid peroxidation and protein oxidation [30]. Resistin concentrations have been also associated with markers of oxidative stress in healthy individuals [31], patients with type 2 diabetes [32], and in patients undergoing cardiac surgery [33]. Carnosine may therefore improve adipokine concentrations via reducing oxidative stress, and further studies investigating this mechanism are warranted.

A second possible mechanism is the commonly reported effects of carnosine on advanced glycation end products (AGEs) [10]. Carnosine has been shown to prevent formation of AGEs and ALEs and restrict any further glycation by both a non-enzymatic glycation and by a direct reaction with reactive carbonyl species generated by lipid and sugar oxidation [34,35,36,37]. Studies have demonstrated that AGEs/ALEs can affect the cellular function of adipocytes, particularly the synthesis and secretion of adipokines [38,39]. In non-diabetic obese individuals, 4-hydroxynonenal, the main reactive carbonyl species target of carnosine, was positively correlated with leptin [40]. AGE concentrations were positively and independently associated with leptin concentrations in patients with type 2 diabetes [41]. Similarly, serum resistin concentrations were associated with soluble receptor for AGEs [42], suggesting a potential link between resistin concentrations and AGEs. Hence, carnosine supplementation may influence AGEs content in adipocytes, thereby regulating the synthesis and production of adipokines.

Finally, the anti-inflammatory properties of carnosine may also play a role in improving adipokine concentrations. In vivo, administration of carnosine suppressed activation of nuclear factor *kappa*-B (NF-κB), a transcription factor with a key role in inflammation and immunoregulation [43]. It has been shown that the effects of both resistin [44] and leptin [45] are mediated by NF-κB activation. Adipokines are also involved in the regulation of inflammatory responses. This is supported by studies which reported positive associations between adipokines and markers of inflammation [46,47]. In morbidly obese individuals [46] and in dialysis patients [48], leptin concentrations were positively correlated with concentrations of inflammatory markers including circulating CRP and tumor necrosis factor. Serum resistin concentrations were also directly associated with markers of inflammation in obese people [49] and post-menopausal women with rheumatoid arthritis [50]. However, we found no associations between hsCRP and the adipokines measured in our study, and no effect of carnosine on hsCRP concentrations [16], which is likely due to our small sample size and low concentrations of CRP in our population. Nevertheless, it is possible that carnosine may regulate the release of adipokines through inhibiting NF-κB activation and reducing inflammation markers, and this should be further explored in larger intervention trials with carnosine supplementation. 

This clinical trial has several strengths, including the use of rigorous methodology and a double-blind randomized placebo-controlled design. This was the first study examining the effects of carnosine supplementation on adipokine concentrations such as resistin, leptin and adipsin in humans. The study participants were metabolically well-characterized, where there was no confounding by disease status or medication use and no differences between groups at baseline. The main limitation of this study was the small sample size as this was a pilot study, where adipokines were not the primary outcome and there was no formal power calculation for adipokines. Further larger trials are needed to confirm the effect of carnosine on adipokines. Moreover, participants in this study were overweight or obese, non-diabetic adults, hence our results may not be generalizable to other populations including lean adults or those with existing diseases.

## 5. Conclusions

We have shown for the first time that carnosine supplementation reduced serum resistin and leptin concentrations in overweight or obese, otherwise healthy adults. These findings indicate the potential role of carnosine in the prevention and treatment of obesity-related cardiometabolic diseases through reducing serum adipokines; however, further studies with larger sample sizes are needed to confirm these preliminary results.

## Figures and Tables

**Table 1:** Effect of carnosine supplementation on serum adipokine concentrations.

Parameters	Carnosine Group (*n* = 13)				Placebo Group (*n* = 9)				Change Difference	*p* #	*p* ^¶^
	Baseline	Follow up	*p* *	Change	Baseline	Follow up	*p* *	Change	Mean ± SE		
Adipsin (ug/mL)	5.22 ± 4.30	2.61 ± 0.79	0.15	−2.60 ± 4.51	2.89 ± 2.03	3.61 ± 3.57	0.67	0.72 ± 3.82	−3.33 ± 1.84	0.13	0.17
Leptin (ng/mL)	114.97 ± 146.45	38.75 ± 40.42	0.14	−76.21 ± 165.69	34.60 ± 52.22	70.12 ± 55.74	0.07	35.52 ± 55.01	−111.74 ± 57.66	0.11	0.06
Resistin (ng/mL)	55.67 ± 75.49	19.86 ± 20.43	0.34	−35.81 ± 83.28	14.41 ± 7.09	34.75 ± 34.55	0.04	20.34 ± 28.97	−56.15 ± 29.08	0.15	0.04

SE, standard error; *p*, *p*-value. Means and standard deviations are reported. * Wilcoxon sign-rank tests for differences between baseline and follow up within groups. ^#^ Mann–Whitney tests for differences between groups at baseline. ^¶^ Mann–Whitney tests for differences in mean change between groups.

**Table 2:** Multivariable quantile regression analyses for relationships between change in serum adipokines and change in urinary carnosine levels.

Dependant Variable	Leptin (ng/mL)			Resistin (ng/mL)		
	β	95% CI	*p*	β	95% CI	*p*
Urinary carnosine (nmol/mL)	−2.27	−3.38, −0.17	0.001	−1.52	−1.85, −1.18	<0.001
Carnosine-propanal (nmol/mL)	−69.71	−126.46, −12.96	0.01	−24.01	−48.95, 0.92	0.05
Carnosine-propanol (nmol/mL)	−67.96	−120.41, −15.51	0.01	−7.93	−19.02, 3.15	0.14

β, beta-coefficient; CI: confidence interval. Regression model: Relationship between change in leptin/resistin and change in urinary carnosine or carnosine adducts after adjusting for age, sex, change in body mass index and intervention group.
